# Wavelength Selective Solar Cells Using Triple Cation Perovskite

**DOI:** 10.3390/nano12193299

**Published:** 2022-09-22

**Authors:** Ahmed Hayali, Roger J. Reeves, Maan M. Alkaisi

**Affiliations:** 1 Department of Electrical and Computer Engineering, University of Canterbury, Christchurch 8041, New Zealand; 2 The MacDiarmid Institute for Advanced Materials and Nanotechnology, Wellington 6140, New Zealand; 3 School of Chemical and Physical Sciences, University of Canterbury, Christchurch 8041, New Zealand

**Keywords:** wavelength selectivity, cesium lead halide, solar cells, triple-cation perovskite

## Abstract

Perovskite materials offer high-efficiency low-cost solar cells and applications versatility. We report on cesium-based hybrid perovskite solar cells with wavelength-selective properties ranging from 500 nm (UV-VIS) to 800 nm (IR). The band gap tuning was achieved through composition changes of mainly lead(II) iodide PbI_2_ and lead(II) bromide PbBr_2._ The optical spectra of the developed materials were studied, including the photoluminescence (PL), optical transparency, X-ray diffraction and external quantum efficiency for samples prepared under different compositions. It was found that a high content of iodine displayed a photoluminescence (PL) peak at 790 nm, whereas a high content of bromine showed a PL peak at 548 nm. The combined composition mixture of PbI_2_ and PbBr_2_ can be fine-tuned to prepare materials that absorbed light in the visible range (640–660 nm) or other selective wavelengths in the range from 500 to 800 nm. The illuminated current-voltage characteristics of the solar cells were carried out under the AM 1.5 condition using an ABET solar simulator with a reference solar cell for comparison and control. The average efficiency of the fabricated solar cells ranged from 3.5% to 15.5%, depending on perovskite composition. Wavelength-selective solar cells have potential applications in smart windows, building of integrated PVs and solar-operated greenhouses.

## 1. Introduction

Due to the global climate changes and energy demands, renewable energy sources are proposed to provide the electricity in a secure and sustainable manner. Moreover, energy supply for food production has to be provided for large-scale production structures such as greenhouses. Generally, light is one of the important factors in the plant’s growth cycle and is affected by two main factors: intensity and radiation spectral distribution of light. The photosynthesis process, for example, depends more on intensity of light energy, whereas plant taste, colour and aroma can be affected by the radiation spectral distribution of light [1]. The commonly used environment of growing plants for mass production under controlled conditions including the light intensity are greenhouses. However, electricity is required for irrigation, ventilation, sensing, heating and lighting. As a result, plants growing in greenhouses are more expensive. To reduce the cost of plantation, solar cell-powered greenhouses are proposed. Thin-film amorphous silicon solar cells were reported recently for greenhouse applications [2]. However, it has been found that thin-film amorphous silicon absorbs light intensity wavelengths in the range between 500 and 600 nm that are fundamental for plant photosynthesis [3]. Thus, to optimise the light available for plant growth, some wavelength tunings of glasshouse structures are required. Nanoclusters or nanoparticles with different sizes can be used to achieve wavelength selectivity and tuneable band gaps. However, their fabrication is still challenging especially for mass production processes such as solar cell fabrication [4].

In this article, development of perovskite-based solar cells that have the potential for greenhouse applications is presented. An attempt to develop solar cells that share the sunlight with plants through wavelength selective properties has been proposed in this work. We demonstrated achieving wavelength selectivity by changing the composition of triple-cation perovskite thin films. The photoluminescence (PL) peak has been shifted by changing anion-like iodine (I), bromine (Br) and chlorine (Cl) concentrations in I/Br, Br/Cl and I/Cl mixtures [5]. We reported on perovskite solar cells PvSCs with selective wavelengths responses that enable the fabrication of solar cells that absorb wavelengths, which are not utilised by plants or not desired in certain glass building structures. In other words, they are solar cells that share the sunlight with other absorbers. This has the potential of opening up new applications of perovskite-based solar cells in smart windows, building of integrated PVs and greenhouses.

Different concentrations of an I and Br mixture with methylammonium (MA) was reported by Noh et al. in 2013 [6]. They have found that increasing the content of Br in the perovskite active layer (MAPbI_3_) has led to a shift towards blue wavelengths of the optical absorption spectra. Kulkarni et al. in 2014 have shown the possibility of achieving a bandgap tuning of lead halide PvSCs [7]. Another attempt in 2015 to use reversible halide exchange reaction as an alternative tuning method has been reported by Jang et al. [8]. Other researchers have achieved high visible light absorption and fabricated semi-transparent PvSCs with transparent electrodes [9,10]. It was also reported that the use of an anti-solvent in the preparation process of perovskite could improve its transparency [11]. A number of studies have focused on wavelength adjustment by adding another layer on perovskite active materials or combining the perovskite cell with other devices [12,13,14]. Others have proposed that by changing iodine, bromine, or chlorine in MAPbX_3_ mixtures, bandgap tuning can be achieved and resulted in a shift in the absorption spectrum [15]. Although other researchers reported on the bandgap tuning, these were not specifically for greenhouse or building of integrated PV applications. In our study, we prepared a range of perovskite-based solar cells that can absorb the sunlight with a wide range of the absorbance spectrum by changing the composition of the perovskite film. 

To our knowledge, there is little research reported on the use of triple-cation perovskite to achieve wavelength selectivity, and no study has been reported on changing the composition of the cesium-based hybrid perovskite layer to achieve materials with a selective wavelength absorption. There is one study that utilised triple-cation mixed-halide perovskites to tune the bandgap for laser purposes [16]. However, the reported literature has not shown a full set of PL characterisation and optical properties for triple-cation perovskite solar cells. In the present study, we reported on perovskite films and solar cells prepared with different compositions of cesium with detailed optical characterisation and devices performance analysis. These devices exhibited efficiencies ranging from 3.5% to 15.5% and bandgaps ranging from 1.52 eV to 2.3 eV. The optical transmissions, photoluminescences, PL decay times, EQEs and XRD spectra for different composition are presented and discussed. The efficiency of solar cells prepared was measured for each composition, and full device characterisation including series, shunt resistances and fill factors were recorded. The active area of all devices studied in the present work were 0.36 cm^2^ with the highest efficiency achieved of 15.5%. The active area of 0.36 cm^2^ was higher than what was typically reported in the literature.

## 2. Experimental Work

### 2.1. Materials

Commercially supplied FTO glass substrates with conductive Soda Lime (FTO, 12–15 Ω/sq) from MSE pro^TM^ company (Tucson, AZ, USA) were employed in all samples used in this study. The main standard reference composition of this study was denoted as sample 1. The composition of sample 1 contained formamidinium iodide (FAI), methylammonium bromide (MABr), lead(II) iodide (PbI_2_) and lead(II) bromide (PbBr_2_) with concentrations presented in Table 1. Following that, changing the compositions of all other materials was attempted to obtain a shift in wavelength and a change in bandgap. Formamidinium bromide (FABr), formamidinium chloride (FACl), methylammonium iodide (MAI) and methylammonium chloride (MACl) were changed to achieve wavelength selectivity. All these chemical materials were procured from Greatcell solar. The concentrations of lead(II) iodide (PbI_2_), lead(II) bromide (PbBr_2_), lead(II) chloride (PbCl_2_), cesium iodide (CsI), cesium bromide (CsBr) and cesium chloride (CsCl), which were purchased from Luminescence Technology Corp. (Lumtec) (New Taipei City, Taiwan), were varied to achieve wavelength selectivity. 

A hole transport layer was fabricated using spiro-MeOTAD 99% (HPLC), tris(2-(1H-pyrazol-1-yl)-4-tert-utylpyridine) cobalt(III) tri(bis(trifluoromethane)sulfonyl)imide) FK209 Co(III) TFSI salt, bis(trifluoromethane) sulfonimide lithium salt (Li-TFSI) 99.9%, acetonitrile anhydrous, (99.8%), 4-tert-Butylpyridine (TBP) 98% and chlorobenzene (95%), which were bought from Sigma-Aldrich (Darmstadt, Germany). The concentrations used in this study to fabricate the perovskite active layer are shown in Table 1. 

### 2.2. Solar Cell Fabrication

In this study, the electron transport layer (ETL) and the hole transport layer (HTL) were kept the same for all solar cell structures prepared. Perovskite thin films were prepared with varied compositions to achieve wavelength selectivity. A commercial FTO glass substrate was etched on one side by zinc powder and HCL acid (2 M) diluted in DI water to avoid a short circuit during the test of the cell. Then, the substrates were cleaned with acetone, methanol and IPA for 10, 5 and 5 min, respectively. They were then dried with N_2_ gas and left in an oven at 100 °C for 40 min. Plasma ashing was used to improve the adhesion and remove any residual organic particles on the FTO glass. This was followed by deposition of 70 nm-thickness compact TiO_2_ on the FTO glass substrate using DC sputtering powered at 200 W [17]. One hundred and fifty milligrams of mesoporous TiO_2_ paste (30NR-D) were dissolved in l mL of ethanol and stirred at 70 °C for at least 3 h before use. This was followed by the deposition of M-TiO_2_ on the compact TiO_2_. The M-TiO_2_ was doped with FK209 (2.5 mg dissolved by 200 µL acetonitrile). A 150 nm thick M-TiO_2_ was achieved using a spinner at a speed of 4000 rpm with a 2000 rpm/s acceleration for a duration of 10 s. The M-TiO_2_ layer was annealed at 450 °C for half an hour. 

An active perovskite layer was prepared as illustrated in Table 1. A perovskite layer was deposited inside a glove box (filled with N_2_) by using two steps. The first step was pouring 100 µL of the triple-cation perovskite on the substrates subjected to a spinning speed of 1000 rpm for 10 s. Then, a second step of spinning at 6000 rpm for 25 s followed by dispensing 230µL chlorobenzene 10 sec before the end of the rotation and then annealing at 100 °C for 40 min was performed to remove the solvent and improve the crystal quality of the perovskite layer [18].

**Figure 1:**
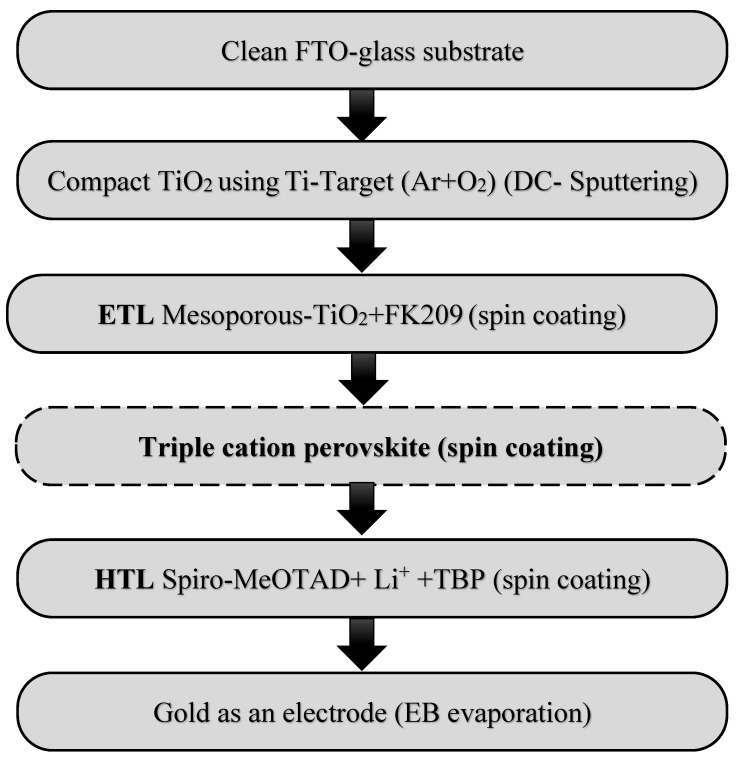
A flowchart of perovskite solar cell manufacturing.

For the hole transport layer, 72 mg of spiro-MeOTAD were first dissolved with 1 mL chlorobenzene. Then, 29 µL of 4-tert-Butylpyridine and 18 µL of Li salt were added to the spiro-MeOTAD. This was accomplished by using 520 mg of lithium salt bis(trifluoromethane) sulfonimide dissolved in 1 mL of acetonitrile. Finally, 70 nm of gold were deposited on the cell as the electrodes using electron beam evaporation. Figure 1 illustrates the flowchart of the perovskite solar cell manufacturing process. 

### 2.3. Characterisation

The surface morphologies of the perovskite active layer with different compositions were imaged using a scanning electron microscope (SEM) based on a Raith-150 EBL machine (Dortmund, Germany). The relationships between optical transmission and wavelengths were measured using a Cary 6000i ultraviolet-visible spectrometer. All current−voltage (J−V) characteristics were measured using ABET Sun3000 with AM 1.5 G sunlight simulator conditions providing an average light intensity of 100 mW/cm^2^. Photoluminescence spectrometries and PL decay times of different compositions were analysed at room temperature using a pulsed 1 mW diode laser at a wavelength of 405 nm. Emission spectra were recoded with an Ocean Optics HR4000 UV-NIR fibre-fed spectrometer. PL decay transients were recorded with a Hamamatsu H8259-01 PMT filtered with selective glass filters. A Stanford Research Systems SR430 photon-counting averager processed the signals. An ABET silicon solar cell was employed for comparison and reference calibration. X-ray diffraction (XRD) based on a Rigaku SmartLab X-ray diffractometer system was used to characterise the crystal structures of perovskite films with different compositions. 

Discrete optical wavelengths for a spectral response (SR) measurement were obtained from the transmission of a 100 W quartz iodine lamp by a small Bausch and Lomb monochromator. The bandwidth (FWHM) of the light was 25 nm. The optical power at each wavelength was measured by a Newport model 818-SL detector head and a model 1916-R metre. The photocurrent at each wavelength was measured on a Keithley model 2000 multimetre. Care was taken to ensure that a consistent sample/illumination geometry was obtained between samples, and measurements were the average of several iterations. The photocurrent (in amps) and the optical power (watts) determined the SR (A/W) that was converted to external quantum efficiency (EQE) by Equation (1):

(1) $$EQE=SR\frac{hc}{\lambda q},\;$$

where *λ* is the wavelength of the light; *q* is the electron charge of 1.6021 × 10^−19^ C; *h* is the Planck’s constant of 6.63 × 10^−34^ J∙S; *c* is the light speed of 2.997 × 10^8^ m/s.

### 2.4. Modification of Perovskite Composition

**Figure 2:**
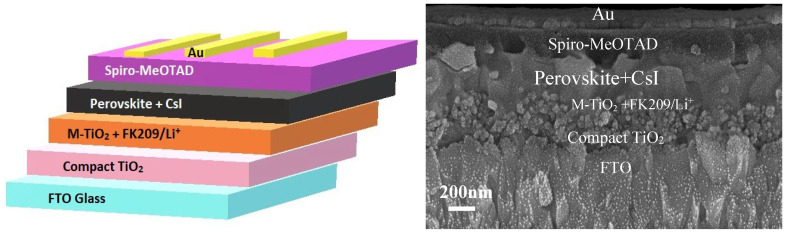
(**Left**) A schematic structure of the perovskite solar cell; (**right**) an SEM image of the cross-sections of the perovskite solar cell device fabricated in this study.

Table 1 shows all the compositions of the triple-cation perovskite examined in this work. The devices fabricated for this study were based on an n-i-p mesoscopic structure with triple-cation perovskite utilised as the active layer. The left of Figure 2 illustrates the main structure of the PvSC device employed in this work, and on the right, it shows an SEM image of the cross-sections of the different layers of the whole PvSC structure. This allowed verification of the thickness, actual structure of each layer and the interfaces between the different layers. The perovskite solar cell (sample 1) comprised a 70 nm thick C-TiO_2_ layer and a 150 nm thick M-TiO_2_ doped with cobalt FK209. The active perovskite layer thickness was 350 nm, followed by a 180 nm thick Spiro-MeOTAD, and finally, 70 nm of the electron beam evaporated gold as the backside electrode for the complete solar cell device. 

This study investigated changing the composition of the perovskite active layer to achieve wavelength selectivity while keeping all other device layers the same. All the modifications in the composition were performed on the triple-cation perovskite active layer, as this material produced the highest efficiency in our work.

The electron transport and hole transport layers were unchanged for all samples in this work. Table 1 lists the five compositions employed and shows the molar ratio of each chemical material and the formula description of each composition. For example, composition 1 (CsI)_0.05_[FAMAPb(I_0.85_Br_0.15_)_3_]_0.95_ was the control composition sample, which was obtained by mixing 1 M of FAI, 0.2 M of MABr, 1.1 M of PbI_2_ and 0.2 M of PbBr_2_ and finally adding 5% of CsI to the total precursor volume. In sample composition 2, a high content of iodine was obtained by mixing FAI (1 M), MAI (0.2 M) and PbI_2_ (1.3 M) and then 5% of CsI added to the total precursor volume. In sample composition 3, a high content of bromine with a low content of iodine was obtained by using FABr (1 M), MAI (0.2 M), PbI_2_ (0.2 M) and PbBr_2_ (1.1 M) and then 5% of CsBr added to the total precursor volume. In sample composition 4, a high content of bromine with a low content of chlorine was obtained by using FABr (1 M), MACl (0.2 M), PbBr_2_ (1.1 M) and PbCl_2_ (0.2 M) and then 5% of CsBr added to the entire precursor volume. Finally, sample composition 5 was obtained by using FAI (1 M), MABr (0.2 M), PbI_2_ (0.63 M) and PbBr_2_ (0.79 M) and then 5% of CsI added to the total precursor volume.

## 3. Results and Discussion

### 3.1. Optical Properties of Various Compositions of the Perovskite Solar Cell

**Figure 3:**
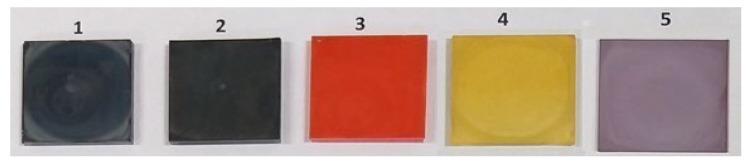
Optical images of the perovskite active layers with various compositions deposited on TiO_2_-coated glass.

The optical images of the bare perovskite films are shown in Figure 3, and it is apparent that significant differences in colour resulted from the mixed anion compositions. 

**Figure 4:**
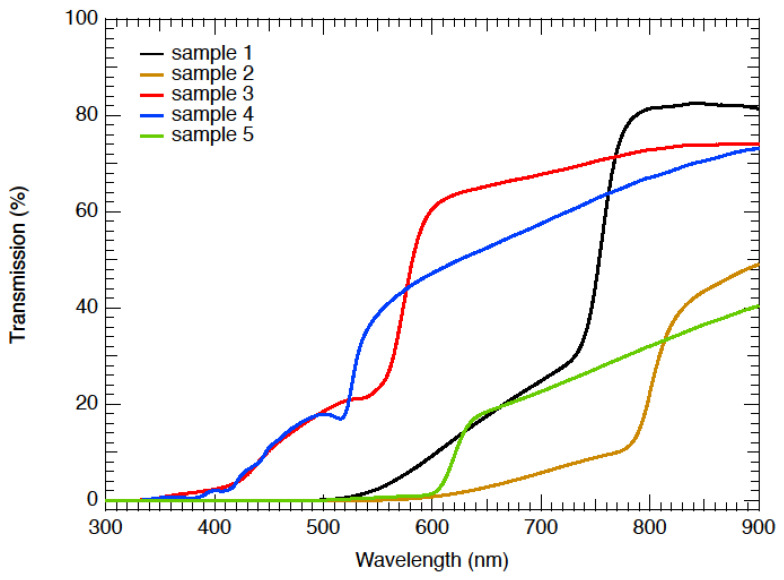
Optical transmissions of the perovskite films with varying anion compositions.

Quantifying these optical changes was performed with transmission spectra, which are shown in Figure 4. All samples showed a distinct edge to the transmission, indicative of an optical bandgap that changed with anion composition [19].

It is apparent that the visible transparency window increased (blue-shift of the absorption edge) as the % of Br relative to I increased, as shown in ascending order of the follows: sample 2 (Br/I = 0), sample 1 (Br/I = 0.20), sample 5 (Br/I = 1) and sample 3 (Br/I = 4.9). This is consistent with the previous literature [5,20] and results from the contraction of the lattice when bromide (radius: 1.96 Å) replaced iodide (radius: 2.19 Å). The same consistent trend occurred, when smaller chloride particles (radius: 1.81 Å) replaced iodide for samples 4 and 3.

**Figure 5:**
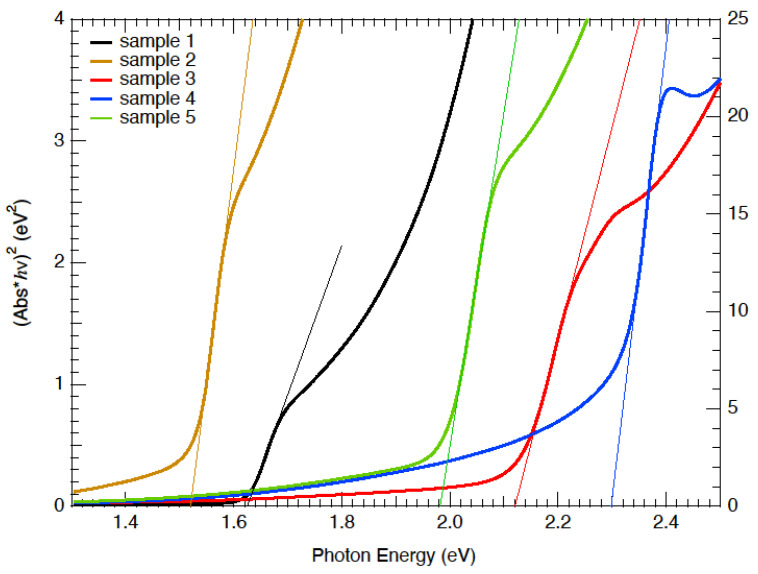
Tauc plots, assuming a direct bandgap, of the solar-cell devices with varying anion compositions.

The optical bandgap can be obtained from a Tauc analysis [21] of the absorbance data (absorbance = −log(T)) with the results shown in Figure 5.

**Figure 6:**
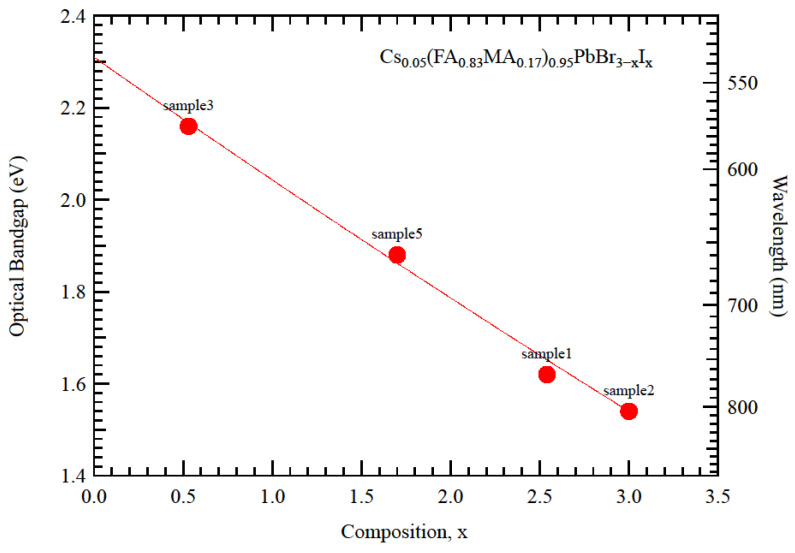
Optical bandgaps for the mixed Br/I compositions.

The pure iodide compound (sample 2) had a bandgap of 1.52 eV, which lied between the values of pure MAPbI3 (1.57 eV) and FAPbI3 (1.48 eV) [5]. Clearly, the small amount of CsPbI3 (Eg = 1.73 eV) widened the bandgap beyond a simple proportionality calculation. Figure 6 shows the optical bandgaps for the mixed Br/I compositions. The data spanned over a wide composition range, indicating that a desired transmission edge can be obtained through the visible spectrum.

### 3.2. Photoluminescence Measurements of Various Compositions of the Perovskite Films

**Figure 7:**
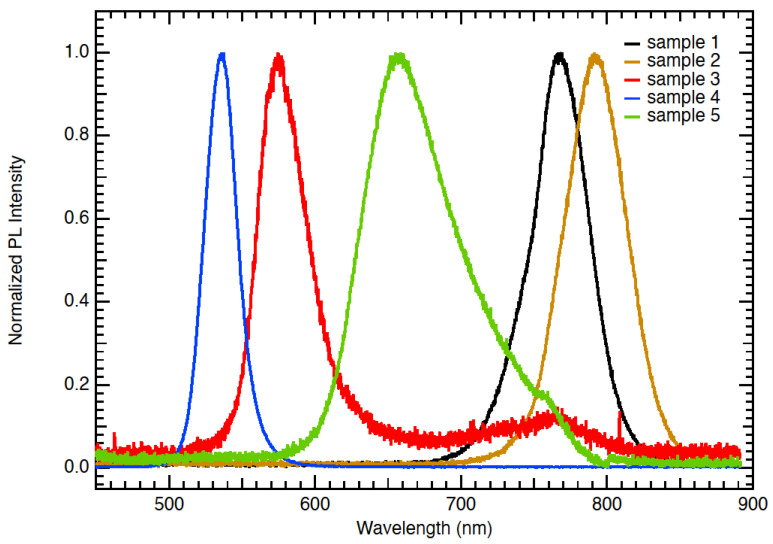
Normalised photoluminescence peaks of various compositions of the perovskite material. Group A (IR range) is represented by samples 1 and 2. Group B (UV range) is represented by samples 3 and 4. Group C (VIS range) is represented by sample 5. Sample 5 exhibited an emission peak at 655 nm, illustrating the PL peak emission can be controlled by composition changes.

Figure 7 shows the photoluminescence (PL) spectra of the five materials under study. Indicated in the figure is the absorption edge determined for each sample. The small Stokes shift between the PL peak and the absorption edge indicated band-to-band (excitonic) transitions rather than mid-gap defect states. PL measurements were conducted to extract the PL peak emission for different compositions of the perovskite active layer. Figure 7 illustrates the normalised photoluminescence spectra for various compositions of the perovskite material. 

We may group the materials according to their PL peaks, group A (IR range) containing samples 1 and 2 had high contents of iodine with the PL peak emission from 770 nm to 790 nm. By increasing the concentration of bromine and decreasing the concentration of iodine, the PL peak was shifted to the blue short-wavelength region of the spectra. Group B (UV range) is represented by samples 3 and 4 with a peak PL emission at a wavelength range from 525 nm to 575 nm. For this particular sample 3, we observed a higher-than-normal noise at 750–775 nm wavelengths. This could be due to variation in its thickness compared to the others. Group C (VIS range) represented by sample 5 (with equal concentrations of iodine and bromine) produced a PL peak at 655 nm. Sample 5 exhibited an emission peak at 655 nm, illustrating the PL peak emission can be controlled by composition changes. The PL spectra of the samples tested in this study are in agreement with what have been reported in the literature [5,22,23].

**Figure 8:**
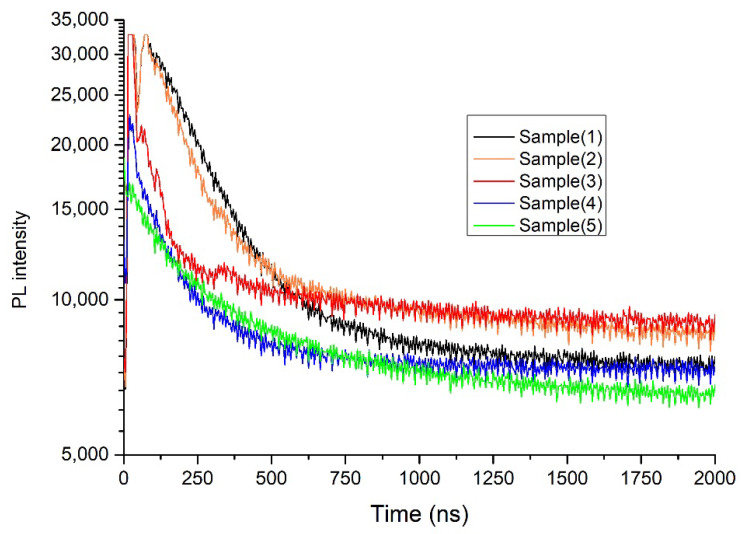
Photoluminescence intensity decay lifetimes of various compositions of the perovskite materials.

Figure 8 shows the decay lifetimes of PL intensity for different perovskite compositions. The lifetime values were determined using fitted decay parameters. These measurements were made on perovskite films only without hole or electron quenching layers.

The obtained results showed that sample composition 1 with a high content of iodine had a long lifetime of 174 ns compared to sample 3 of high content of bromine, which had a short lifetime of 96 ns. Sample 5 (with equal concentrations of iodine and bromine) had a lifetime of 142 ns. A high carrier lifetime of perovskite indicates enough time to separate electron-hole pairs before recombination, which can improve the performance of PvSCs [24].

### 3.3. Structural Properties of Various Compositions of the Perovskite Films

**Figure 9:**
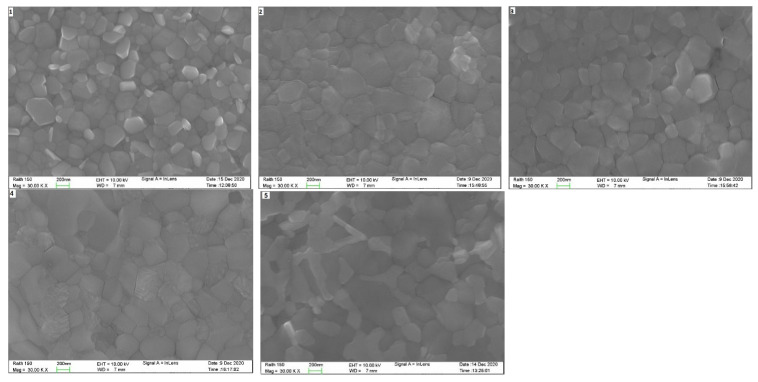
SEM images of the perovskite active layers prepared with different compositions for the 5 samples as detailed in Table 1 and are numbered accordingly.

Figure 9 displays direct incident SEM images of the five main different active perovskite layers prepared with different compositions. Different compositions of the triple-cation perovskite films resulted in different grain sizes and crystal structures. 

Table 2 lists the average solar cells efficiencies and the corresponding bandgaps for the five samples of perovskite films prepared with different compositions. Generally, we did not find the correlation between the band gap energy value and the efficiency of perovskite cells due to other factors such as absorption, transparency, grains size and series resistance, which might affect the efficiency. For example, in composition 3, the energy gap was 2.12 eV, but it had also a high transparency, which allowed most of the incident light to pass through the active perovskite layer and not to be absorbed. Moreover, this composition had a shorter lifetime compared to other compositions. The SEM image of this sample showed cracks in the perovskite film structure, which might lead to a high series resistance and resulted in a drop in efficiency. There were correlations between the PL lifetime and the efficiency of different perovskite compositions. For example, sample composition 1 had a higher efficiency and a longer lifetime. A long lifetime of perovskite indicates enough time to generate photogenerated electron-hole pairs and separate them before recombination occurs, which results in improving the performance of PvSCs [24].

**Figure 10:**
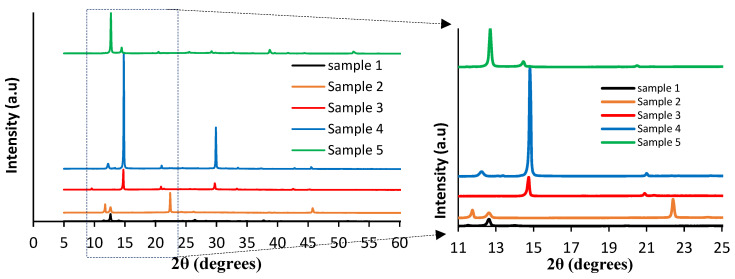
(**Left**) XRD patterns spectra of the perovskite active layers prepared with different composition; (**right**) XRD patterns focused on the range of 11° to 16°.

X-ray diffraction (XRD) analysis was conducted on different compositions of triple-cation perovskite prepared on the FTO glass substrate. The XRD was used to characterise and analyse the crystal structure (crystallographic properties) of the perovskite active layer. Figure 10 shows XRD spectra of perovskite active layers with different compositions. Sample 1 (a high content of iodine and a low content of bromine) exhibited a low XRD intensity at an angle of 12.7°. In sample 2 (a high content of iodine), the first peak observed was at 2θ of 11.8°, and the second peak was at 22.5°. Sample 1 exhibited the typical characteristic perovskite XRD peak at 11.8°, which agrees with most reported XRD studies [25,26,27]. Sample 3 (a high content of bromine and a low content of iodine) had an angle of 14.7°, and sample 4 (a high content of bromine and a low content of chlorine) had an angle of 14.8° [20]. Sample 4 produced the highest XRD intensity. Suzuki et al. has shown that by increasing the percentage of Br, the lattice constant of the bromine ions is decreased [28]. It can be observed that when equal concentrations of iodine and bromine were used (sample 5), the intensity was higher as compared to sample 1, but both produced a peak intensity at 2θ of 12.7°. Shifting the value of 2θ from low to high meant that the lattice constants were decreased. This is because of the lattice decrease in size; in this case, the shift was towards a larger angle. If the lattice increased in size, it shifted towards a smaller angle. This is an illustration that how the crystal structure and lattice constants of the perovskite film were changed by changing the composition. The use of iodine, therefore, reduced the value of the 2θ angle, and the use of bromine or chlorine increased the 2θ angle.

### 3.4. Current−Voltage Characteristics of Various Compositions of the Perovskite Solar Cell

**Figure 11:**
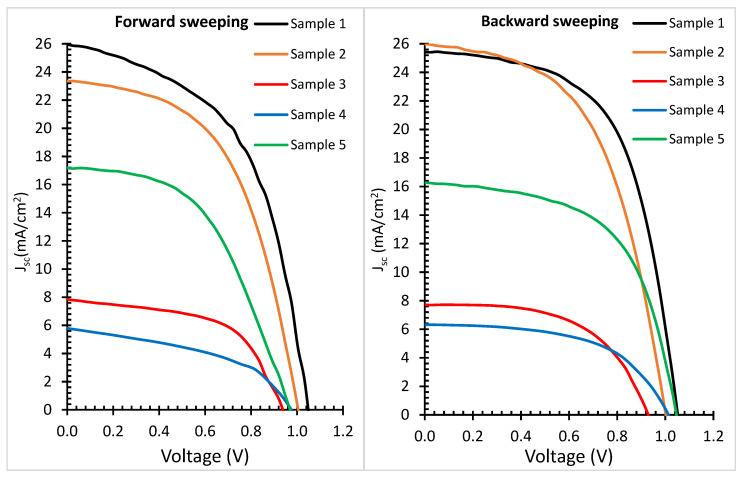
J−V characteristic curves of various compositions of the 5 perovskite solar cells: (**left**) forward sweeping; (**right**) reverse sweeping. The scan rate was 0.028 V/s.

Figure 11 shows the current density−voltage (J−V) characteristic curves of the PvSCs prepared with different compositions for both the forward and backward sweeping. In this work, the overall samples size was 2.5 × 2.5 cm^2^ with an active device area of 0.36 cm^2^. Five samples were fabricated in the same batch for each composition to verify the reproducibility and uniformity. It can be seen from Table 3 that by changing the composition of the perovskite active layer, the performances of the devices varied due to changing of the bandgap, absorption peak, lifetime and film conductivity. The hysteresis index was measured using the equation below:

(2) $$\mathrm{Hysteresis}\;\mathrm{index}\;\left(H.I\right)=\frac{\mathrm{PCE}\;\left(B.w\right)-\mathrm{PCE}\;\left(F.W\right)}{\mathrm{PCE}\;\left(B.w\right)},$$

where PCE (B.w) is the power conversion efficiency for backward scanning and the PCE (F.W) is the power conversion efficiency for forward scanning. The hysteresis indices which was caused by charge redistribution during the J−V measurements are shown in Table 3 for samples 1 to 5.

Both forward and backward sweeping were tested, and the results are shown in Table 3. Generally, it can be observed from Table 3 that high-efficiency perovskite solar cells were achieved when a high content of iodine and a low content of bromine were used as demonstrated by sample 1 testing results. Using a high content of bromine and a low content of iodine or chlorine resulted in a drop in the efficiency of the PvSCs to around 4%. The J_sc_ value was dependent on a number of factors including the final device structure. The J_sc_ varied with perovskite composition, the energy gap of the material and the optical and electrical properties of each layer. Each layer was also influenced by the interfaces between the various layers. For example, in sample 1, the perovskite film was deposited on TiO_2_, which might result in different structures compared to sample 2 because of the grain size dependence. The different values of J_sc_ were attributed also to different conductivities, transparencies and mobilities of the device layers, in addition to the fact that different samples had different perovskite compositions. It is worth mentioning that we have prepared perovskite solar cells that had peak absorption at 600–700 nm wavelengths. This was achieved by using equal concentrations of iodine and bromine as in sample 5, and the efficiency was found to be around 9.5%.

**Figure 12:**
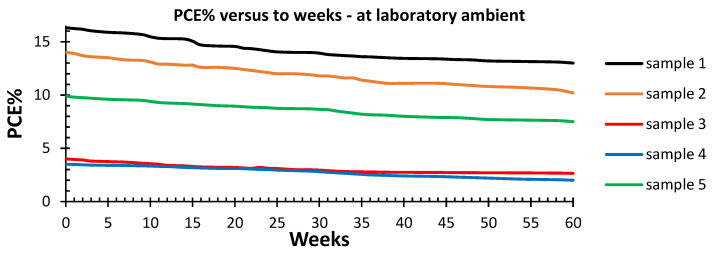
Devices stability testing results by measuring variations in the efficiency of the perovskite solar cells over a 60-week period. Devices were kept in an ambient laboratory condition and tested under a 100 mW/cm^2^ illumination using ABET AM 1.5 G sunlight simulators with a reference cell as a control.

**Figure 13:**
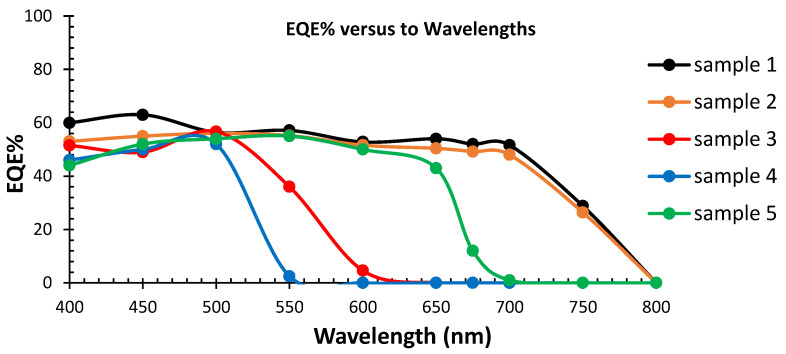
External quantum efficiency spectra of the perovskite solar cells prepared with different compositions.

The stability was monitored for all devices. The stabilities of samples 1 to 5 were monitored through their conversion efficiency measurements and were conducted over 60 weeks under laboratory ambient conditions of a 50% humidity and a temperature of 25 °C. Ten samples were tested for stability and reproducibility studies, and the results are shown in Figure 12. Sample1 exhibited a 19% drop in efficiency as compared to sample 2, which showed a drop in efficiency of 27%. The drops in the efficiency for samples 3 and 4 were around 40%. Sample 5 exhibited a drop in efficiency of 24%. This is in general agreement with the reported stability observed in perovskite-based solar cells. However, a detailed stability study is beyond the scope of this work. Figure 13 shows the external quantum efficiency spectra of the perovskite solar cells with different compositions. The measured EQE varied as expected for samples with different compositions. The EQE graphs for sample 1 and 2 show a continued photocurrent generation at wavelengths up to 800 nm, whereas the photocurrent conversions of samples 3 and 4 dropped at 600 nm and 550 nm, respectively. For sample 5 with a mixed bromine and iodine composition, the photocurrent dropped at 700 nm in agreement with the bandgap value. The EQE data of samples 1 to 5 followed the same trend and changes in the energy gap and the PL response measurements. This further confirmed the wavelength selectivities of these samples.

## 4. Conclusions

Wavelength-selective PvSCs have been prepared, tested, analysed and demonstrated in this work. Bandgap tuning and wavelength selectivity were investigated by changing the composition of the perovskite active layer. The composition of the perovskite active layer played an important role in determining the transmission, lifetime, EQE and photoluminescence spectra. The PL measurement revealed a notable shift in the peak emission of perovskite films when the concentrations of I, Br and Cl were changed. Cesium lead halide perovskite solar cells with wavelength-selective properties ranging from 500 nm (UV-VIS) to 800 nm (IR) have been presented. The bandgap tuning was achieved through composition changes of mainly PbI_2_ and PbBr_2_. The optical spectroscopy of the developed materials was studied, including the photoluminescence, PL decay time, transparency, X-ray diffraction and EQE for samples prepared under different compositions. The bandgap, as measured from PL spectra, exhibited a shift from 1.52 eV to 2.3 eV, when the composition of the perovskite mixture was changed. The PL measurement revealed a notable shift in the peak emission of perovskite films, when the concentrations of I, Br and Cl were changed. Sample 1 with a high content of iodine had a long lifetime of 174 ns compared to sample 3 with a high content of bromine having a lifetime of 96 ns. A shorter carrier lifetime resulted in a lower Jsc, FF, shunt resistance Rsh and subsequently efficiency of the PvSCs. The EQE measurements revealed a cutoff in the photocurrent generation that was dependent on the energy gap of the perovskite material and its composition. An average efficiency of 15.4% have been achieved with the developed PvSCs using a high concentration of I and a low content of Br. The high efficiency was obtained on a 0.36 cm^2^ active area and was measured against a reference certified cell. By using equal concentrations of iodine and bromine (sample 5), an efficiency of around 9.1% has been obtained. The equal mixture produced a material that can absorb light in the visible range with a peak centred at a 660 nm wavelength. The stability of the devices was monitored for over 60 weeks under laboratory ambient conditions. The drop of efficiency with time ranged from 19% to 24%, depending on perovskite composition used. The ability of fine-tuning the bandgap by changing the perovskite composition provides promising opportunities for new applications in building integrated PVs and in greenhouses.

## Figures and Tables

**Table 1:** Compositions of materials used to prepare triple-cation perovskite precursors. The molar ratio of each chemical is shown for each composition.

Sample Composition NO.	Description	FAI (M)	FABr (M)	FACl (M)	MAI (M)	MABr (M)	MACl (M)	PbI_2_ (M)	PbBr_2_ (M)	PbCl_2_ (M)	CsI (%)	CsBr (%)
1	(CsI)_0.05_[FAMAPb(I_0.85_Br_0.15_)_3_]_0.95_	1	-	-	-	0.2	-	1.1	0.2	-	5	-
2	(CsI)_0.05_[FAMAPb(I)_3_]_0.95_	1	-	-	0.2	-	-	1.3	-	-	5	-
3	(CsBr)_0.05_[FAMAPb(Br_0.85_I_0.15_)_3_]_0.95_	-	1	-	0.2	-	-	0.2	1.1	-	-	5
4	(CsBr)_0.05_[FAMAPb(Br_0.85_Cl_0.15_)_3_]_0.95_	-	1	-	-	-	0.2	-	1.1	0.2	-	5
5	(CsI)_0.05_[FAMAPb(I_0.5_Br_0.5_)_3_]_0.95_	1	-	-	-	0.2	-	0.63	0.79	-	5	-

**Table 2:** Efficiencies, grains sizes, lifetimes and bandgaps of perovskite active layers with different compositions.

Sample Composition NO.	Description	Efficiency (%)	Grain Size (nm)	Energy Gap (eV)	Lifetime (ns)
1	(CsI)_0.05_[FAMAPb(I_0.85_Br_0.15_)_3_]_0.95_	15.4	190	1.63	173.99
2	(CsI)_0.05_[FAMAPb(I)_3_]_0.95_	13.3	221	1.52	158.94
3	(CsBr)_0.05_[FAMAPb(Br_0.85_I_0.15_)_3_]_0.95_	4.2	193	2.12	96.28
4	(CsBr)_0.05_[FAMAPb(Br_0.85_Cl_0.15_)_3_]_0.95_	3.5	257	2.3	103.4
5	(CsI)_0.05_[FAMAPb(I_0.5_Br_0.5_)_3_]_0.95_	9.1	249	1.98	142.4

**Table 3:** Perovskite solar cell key parameters measured for different compositions of the perovskite active layer.

Sample Description	Sweep Direction	PCE (%)	H.I (%)	FF (%)	V_oc_ (mV)	J_sc_ (mA/cm^2^)	V_max_ (mV)	J_max_ (mA/cm^2^)	I_sc_ (mA)	R_shunt_ (Ω∙cm^2^)	R_series_ (Ω∙cm^2^)
1	Forward	15	8.5	53	1049	26.4	724	20.3	9.4	1305	9.3
	Backward	16.4		60	1052	25.4	780	20.4	9.07	2024	8.6
2	Forward	12.5	10.7	53	1005	23.3	696	17.9	8.4	975	11.2
	Backward	14		53.9	1003	25.9	696	20	9.3	1725	9.7
3	Forward	4.2	5	56	939	7.8	696	5.94	2.8	532	27.3
	Backward	4		56	928	7.7	668	6.02	2.7	2681	23.2
4	Forward	2.5	28	44.8	973	5.81	691	3.64	2.1	417	39.6
	Backward	3.5		55.3	1013	6.3	754	4.69	2.2	16776	30.7
5	Forward	8.3	16	50	970	17.22	612	13.58	6.14	508	20
	Backward	9.9		58	1046	16.2	780	12.6	5.8	976	11

## Data Availability

Not applicable.
